# Dimension, Content, and Role of Platform Psychological Contract: Based on Online Ride-Hailing Users

**DOI:** 10.3389/fpsyg.2020.02097

**Published:** 2020-09-30

**Authors:** Shengxiang She, Haoran Xu, Zehong Wu, Yunzhang Tian, Zelin Tong

**Affiliations:** ^1^School of Business, Guizhou University of Finance and Economics, Guiyang, China; ^2^School of Business, Guilin University of Technology, Guilin, China; ^3^Center for Behavior and Decision, Shaanxi University of Technology, Hanzhong, China; ^4^Management School, Hainan University, Haikou, China

**Keywords:** online car-hailing platform, relationship quality, value-added validity, psychological contract, psychological contract breach

## Abstract

Online sharing platforms are a new form of enterprising organizations. Their interaction with users exhibits unique characteristics. Based on the extant literature on psychological contracts and interviews, a survey, and statistical analyses of online ride-hailing users, we explore the dimension, content, and role of platform psychological contracts. The results show that the platform psychological contract includes transactional and relational dimensions. The latter dimension features social responsibility contents, which are distinct from that of a traditional enterprise. Using the scale developed herein, we further examine the effect of psychological contract breach on platform relationship quality. Evidently, both dimensions of psychological contract breach are negatively correlated with platform relationship quality. Besides, the value-added validity of relational psychological contract breach with respect to platform relationship quality is higher, suggesting the importance of the relational psychological contract.

## Introduction

The platform economy is a new and valuable force of economic development. Most internationally known brands are now platform-type enterprises. Among vehicle-for-hire services, online ride-hailing platforms Uber, DiDi, and LYFT are predominant in meeting the consumer demand of short-distance traffic^1^. However, these platform enterprises also routinely receive negative publicity for enforcing price discrimination or for retaining contractors accused of sexually assaulting passengers and uncivilized driver behavior. These reports seriously affect user awareness of the service quality and the enterprises’ reputation^2^. According to the user agreement policy of these apps, the legal liability of the platform as a third-party information service provider is limited, although consumers may have a diverse and largely subjective sense of platform responsibility. This difference reflects the inconsistency between the tangible economic and intangible psychological contracts—a challenge to the operation and service management of platform enterprises.

When a consumer purchases a product or service from an enterprise, the resulting exchange constitutes a psychological contract—that is, the consumer’s perception of and beliefs about the enterprise’s obligations (Blancero and Ellram, 1997). At the same time, a service failure event might elicit different levels of dissatisfaction with an online ride-hailing platform because each individual’s psychological contract with the platform varies.

Currently, empirical research on consumer psychological contracts in the platform economy is focused on the relationship between consumers and online businesses (Pavlou and Gefen, 2005; Guo et al., 2015; Malhotra et al., 2017). Although there is some evidence on consumer perception of the responsibility of online platforms, empirical research on the consumer’s psychological contract with platform enterprises is limited. Particularly, extant research does not capture the uniqueness of this contract, causing confusion about the specific contents thereof.

Compared with a traditional enterprise, the platform enterprise, including ride-hailing services, is characterized by diversified market roles, complex influence relationships, and prominent social attributes. More in-depth theoretical investigation on the structure and content of the psychological contract in the platform economy is therefore necessary.

The first purpose of this study is to explore the structure and content of the psychological contract of platform enterprises, develop the scale of the psychological contract, and then test the validity and reliability through standardized procedures. To further verify the validity of the scale, we use brand relationship quality as the criterion to test the predictive ability of psychological contract breach (PCB) with respect to platform relationship quality. Therefore, another purpose of this study is to examine the relationship between PCB and relationship quality.

## Theory

### Origin of Psychological Contracts

The term psychological contract has been widely used in organizational behavior studies to refer to the perceived agreement that exists in the mind of an employee about the employee–employer relationship (Rousseau, 1989, 1995). The employee infers that the particularities of this agreement are made by the employer in return for the employee’s contributions to the organization. Initially, psychological contracts were seen as an individuals’ beliefs about the mutual “give and get” expectations in a relationship (e.g., Levinson et al., 1962; Kotter, 1973). It was later seen as employees’ unidirectional perception of obligations to both parties. Daniel Rousseau redefined it as “an individual’s belief regarding the terms and conditions of a reciprocal exchange agreement between the focal person and another party” (Rousseau, 1989). Roehling (1997) further noted that a psychological contract can be extended to the relationship between an enterprise and consumers outside the organization. For a brand, a psychological contract constitutes a consumer’s perception about the promises made by a brand. These perceived features of the agreement are often unspoken promises that go beyond the tangible and intangible products involved in the exchange. Llewellyn (2001) defined the psychological contract of consumers in B2B contexts as the unspecified agreement between the two parties in the transaction, and the implementation of the agreement is conducive to the smooth progress of the transaction. Montgomery et al.’s (2018) research forms the primary work in marketing on brand–consumer relationships in the context of psychology contracts. They show that committed consumers have psychological contracts with brands, and any violation of any aspect in the contract results in a negative response.

### Concept of Psychological Contracts

Apart from their widely examined economic and legal aspects, contracts also have a psychological component (Macneil, 1980). This component is inherently perceptual and deals with implicit details and perceived obligations that exist beyond those that can be explicitly described in formal legal terms. An economic contract interprets legal commitment, and a psychological contract describes how people understand the commitment terms of both parties. In line with the social exchange theory (Blau, 1964), the perceptual, unwritten, and implicit nature of psychological contracts are their defining attribute (Argyris, 1960), which distinguishes them from legal contracts (Weick, 1979).

In marketing, the incompleteness of contracts means the explicit economic contract between consumers and enterprises cannot include all the responsibilities and obligations between them. Further, consumers are greatly affected by their perceptions of responsibilities and obligations outside the explicit economic contract terms. Therefore, psychological contracts are much broader than economic and legal contracts; they include several perceptual aspects that cannot be formally incorporated into legal contracts.

Psychological contracts in marketing settings are defined as consumers’ perceptions of and beliefs about the implicit and unwritten reciprocal responsibilities and obligations between themselves and enterprises. Some studies have indicated the existence of psychological contracts between buyers and sellers (Pavlou and Gefen, 2005; Kingshott, 2006; Kingshott and Pecotich, 2007; Lövblad and Bantekas, 2010). Compared with consumers’ recognition of their own obligations in a psychological contract, scholars pay more attention to consumers’ recognition of enterprises’ responsibility. In this paper, users’ psychological contracts in platforms are defined as their perceptions of and beliefs about the reciprocal obligations of the platform.

### Dimensions of Psychological Contracts

There are several ways to categorize psychological contracts (Sels et al., 2004; ingshott, 2006). A widely accepted typology views contracts as either transactional or relational (Rousseau, 1995; Rousseau and Tijoriwala, 1998; Pavlou and Gefen, 2005). A transactional psychological contract is based on short-term returns and benefits; a relational psychological contract focuses on general, long-term, social, and emotional connections. The relational psychological contract is an emotional commitment higher than the transactional psychological contract, which is an individual’s recognition of the other party at a higher level of trust.

According to extant research, a psychological contract has two different aspects. For example, when hailing a ride from an online platform, the user spends money and time. Thus, the user takes it for granted that the platform should consider and meet his or her interests, such as a smooth app experience, rapid vehicle arrangement, accurate navigation, and a polite driver. If the platform fails to achieve any of these, the user will feel a breach of the transactional psychological contract, and then be dissatisfied with the transaction. If he or she is a regular user, he or she may have a higher level of identification with the platform based on the experience and trust from past transactions. In this case, the user would develop a relational psychological contract related to the service ability and even the values of the platform. For example, shortly after a negative ride-sharing incident in 2018, DiDi suspended all online ride-hailing services in mainland China from 23:00 to 05:00 in order to implement “safety rehabilitation.” However, this suspension of services led to widespread complaints from users, especially overtime workers who depended on DiDi’s service at night. Some media even questioned whether DiDi was rectifying the problems or using its monopoly to declare war on users^3^.

The above incident is a typical manifestation of the breach of the relational psychological contract. Indeed, users held that DiDi’s suspension of its night service was contrary to its social responsibility. Critics reasoned that because DiDi had become a giant in travel services, it owed users its uninterrupted services. This event shows that the transactional psychological contract is attached to every specific exchange activity, while the relational psychological contract runs through the whole enterprise. Even if no transaction occurs, the relational psychological contract still plays a role in a person’s attitude toward the platform.

Some empirical studies in marketing based on Chinese consumers also divide the psychological contract into transaction and relationship (Luo, 2006; You et al., 2007). However, scholars have argued that the psychological contract has more dimensions. For example, Shapiro and Kessler (2000) proposed three dimensions: transaction responsibility, training responsibility, and relationship responsibility. Kingshott (2006) proposed four aspects in the supplier–buyer context: good faith and fair dealing, intrinsic relational characteristics, relational benefits, and relational conditions. Ma et al. (2013) developed a consumer psychological contract scale reflecting the relationship between shoppers and shopping malls; in their study, they included four dimensions: authenticity and reliability, service environment, service equity, and after-sales service. In the business-to-consumer context, Wang et al.’s (2017) consumer psychological contract scale revealed three dimensions: transaction normative responsibility, service equity responsibility, and relationship development responsibility. Guo et al. (2015) found that the consumer psychological contract in business-to-consumer contexts can be divided into the relational, standard, transitional, and captive types.

Although the above-mentioned multi-dimensional psychological contract is structurally different from the classic two dimensions of transaction and relationship, its content can still be categorized into the two main dimensions.

### Characteristics of Users’ Psychological Contracts With Online Platforms

Scholars mostly focus on traditional marketing and use ordinary service providers (such as hairdressers and shopping malls) to study psychological contracts. However, these developed scales are not applicable to sharing platform enterprises (such as Uber and DiDi) that provide key resources, undertake important functions, and occupy a special position in society. Sharing platform enterprises typically do not directly provide products or services to consumers; they link supply and demand in the market and achieve accurate docking of bilateral users through interface construction, transaction, interaction, and other mechanisms. This makes such services a new type of market organization.

Online ride-hailing platforms have three distinct features: Firstly, the platforms and their users mainly interact through an app, and there is no service contact in real life. Secondly, they provide no direct traffic services; instead, they integrate resources for bilateral users (car owners and passengers) and match them efficiently. Thirdly, with their advanced technology and business model, these platforms have acquired a large amount of traffic and data resources and have become the first choice for most people to travel. To some extent, these platforms have become a kind of social public good. If such a platform were to cease its operations or abuse its status and power, the resulting social effects would be far-reaching. Fourthly, these services are directly related to the personal safety of passengers, which mandates strong ethical attributes and greater social responsibility. Thus, the relationship between users and the platform varies compared with traditional enterprises.

Based on the existing psychological contract theory, this study fully considers the particularity of the online platform service. We not only verify the two-dimensional structure of the psychological contract of the platform user, but also reveal the unique psychological contract content of the user with respect to the online platform.

## Development of Psychological Contract Sale

Mobile broadband has greatly promoted business model innovation. Uber, DiDi, and other online platforms in the travel market provide a low-cost, personalized, and efficient means of hailing rides by integrating a large number of private car resources. These platforms are characterized by high quality, diversity, and differentiation. As a result, they have been enthusiastically embraced by a vast number of users. However, there is still considerable criticism of online car services because of their frequent service failures. Although these failure can be largely attributed to the drivers, public criticism is focused on the enterprises. For example, DiDi, with a market share close to 90% in China and valued at $56 billion by the global database CB Insights, has been criticized by Chinese users for its attitude toward a series of passenger safety incidents.

Owing to the importance of online ride-hailing platforms, the large scale of users, and the representativeness in platform enterprises, we chose this platform to investigate the psychological contract of online ride-hailing users.

### Generation of the Initial Content Scale

Firstly, the initial scale of psychological contracts was designed. We organized a meeting of seven management professionals. Based on the definition of a psychological contract and their own experience using online ride-hailing services, the group proposed the content items of a psychological contract and discussed each item’s suitability. We extracted material from posts and messages from an online forum. Then, interviews were conducted with ten graduate business school students regarding the contents of their psychological contracts with online ride-hailing platforms. Several parts were integrated into an initial questionnaire, which included 32 items.

Next, six marketing and psychology specialists were invited to fill out and check the questionnaire. These experts provided many suggestions on the expressive fluency of the questionnaire, accuracy of the guiding language and item expression, understandability for the general respondents, and overall attractiveness of the questionnaire. After repeated discussion, some items with repeated meanings were merged; as a result, 11 items were deleted to ensure the scale had a higher content validity, and an initial scale including 21 items was finally produced.

### Pilot Survey

Before a formal questionnaire survey, a presurvey was conducted to test the reliability of the scale and the validity of the data. The internal consistency of the survey data and the rationality of the validity test structure were tested through a reliability test of the survey data. The subjects of the test were graduate students (including students pursuing an MBA and a master’s in psychology) in Beijing, Changsha, and Guilin, China. The guiding question to measure the strength of the psychological contract was “To what extent do you agree that the platform has a commitment (or obligation) to do the following,” with a response of “1” indicating “strongly disagree” and “7” indicating “strongly agree.”

A total of 244 questionnaires were collected through the online survey. Among them, 189 questionnaires were valid, and the validity rate of the questionnaires was 77.5%. Based on data analysis of the presurvey, the questions in the items were refined and modified.

We validated the basic structure of the data using exploratory factor analysis (EFA) and conducted factor analysis on the items. The collected data were tested using the Kaiser–Meyer–Olkin (KMO) and Bartlett’s sphericity tests. The KMO value was 0.936, close to 1, and Bartlett’s test had a significant probability of 0; therefore, the scale was suitable for factor analysis.

Then, the principal component analysis method was used to analyze the factors. In previous studies, researchers used the size of factor loadings as the criterion to delete questionnaires. Some researchers, such as Lederer and Sethi (1991), used 0.35 as the critical value, but 0.40 was more common. The factor load corresponding to each item must be close to 1.0, but the factor load corresponding to other factors must be close to 0 (differentiated validity). That is, if the loading of the item is less than 0.40 in all the factors or if there are more than two factor loadings larger than 0.40 (spanning more than two factors), then the item should be deleted. According to this criterion, seven items were deleted.

## Empirical Exploration of the Psychological Contract

### Sample

In total, 480 questionnaires were sent out, and 252 were recovered. Excluding the questionnaires with a very short answering time and concentrated options, 221 questionnaires were ultimately considered valid, with a validity rate of 87.7%. The sample distribution was as follows: 156 female users, accounting for 70.6% of the total sample, and 65 male users, accounting for 29.4%. As the sample was mainly students, respondents aged 18–25 accounted for 96.4% of the total sample, with 81.4% pursuing a bachelor’s degree. Further, 96.4% of the respondents used DiDi over other ride-hailing apps. A total of 149 respondents (67.4%) were frequent users of online ride-hailing platforms, while 68 respondents (30.8%) were occasional users.

We measured the strength of the psychological contract by asking the question “To what extent do you agree that the platform has a commitment (or obligation) to do the following,” with a response of “1” indicating “strongly disagree” and “7” indicating “strongly agree.”

### Item Analysis

Each participant’s scores on each item of the psychological contract questionnaire were summed and sorted according to the level. The first 27% of the total score was the high group, and the last 27% was the low group. The difference of the average scores in each item between the high and low groups was tested. If the difference was significant (*P* < 0.05), it suggested that the item had enough discrimination power and should be retained; if not, it should be deleted. The results of discriminant analysis showed that each item reached a significant level (see *T* value in Table 1), indicating that the items in this scale have the ability to distinguish between high and low groups.

**TABLE 1:** Descriptions of items.

Item	Mean	SD	*T* value	Correlation with total score
Q1. The app is easy to use	5.50	0.966	7.039	0.542
Q2. The app is reliable	5.28	1.088	9.362	0.644
Q3. The fees charged are fair and reasonable	4.97	1.181	9.635	0.660
Q4. The estimations of travel time, distance, and fee are accurate	4.98	1.208	8.245	0.623
Q5. The platform provides accurate navigation and positioning services	5.18	1.150	9.289	0.626
Q6. The platform handles orders efficiently	4.92	1.273	11.172	0.706
Q7. The platform makes reasonable compensation for service failures	4.42	1.477	14.263	0.794
Q8. The platform protects users’ private information	4.44	1.619	15.609	0.798
Q9. The platform does not abuse its market power and information advantage	4.33	1.421	16.846	0.803
Q10. The platform gives priority to social interests	4.22	1.407	13.460	0.740
Q11. The platform ensures legal compliance of drivers and vehicles	4.26	1.599	17.955	0.795
Q12. The platform assumes equal responsibility for all types of cars hailed online	4.36	1.539	15.468	0.784
Q13. The platform is liable to users for any damage	4.38	1.449	12.872	0.724
Q14. The platform attaches importance to continuous improvement and perfection	4.80	1.391	14.633	0.791

Standard deviation describes the average dispersion degree of all the data centered on the mean. A large standard deviation of the item indicates that the subjects’ scores are widely distributed on the item and that the item can identify the differences in individual responses. In contrast, a small standard deviation shows that the subjects’ scores fluctuate within a small range, and the discriminative power of the item is low. Accordingly, items with a standard deviation of less than 0.50 should be excluded. The results show that the standard deviation of all the items in the questionnaire was greater than 0.50 (Table 1), which shows that the items in the questionnaire have good discrimination. The correlation between each item and the total score also reached a significant level of 0.01. The correlation coefficient was between 0.54 and 0.80, which is higher than 0.50, indicating high internal consistency of each item.

### Exploratory Factor Analysis

Bartlett’s sphericity test was conducted on the sample data; the test value was 2008.236 (*p* < 0.000), indicating the possibility of sharing factors among items. The KMO value was 0.913, indicating that the data were suitable for factor analysis. Fourteen items of the psychological contract were analyzed by EFA. After principal component analysis, the factor with an eigenvalue greater than 1 was extracted. Then, the factor analysis results were maximally orthogonally rotated, and two factors were extracted combined with a scree plot (see Table 2). However, the factor loadings of the item “The platform makes reasonable compensation for service failures” on two factors were 0.673 and 0.427, respectively. These values indicate that the item intersects on two factors; as a result, it was deleted.

**TABLE 2:** Factor structure and loadings of the psychological contract.

Item	Factor	
	
	Relational	Transactional
Q1	0.259	0.559
Q2	0.326	0.642
Q3	0.204	0.819
Q4	0.131	0.848
Q5	0.200	0.763
Q6	0.374	0.674
Q7	0.673	0.427
Q8	0.729	0.359
Q9	0.785	0.301
Q10	0.826	0.140
Q11	0.812	0.244
Q12	0.793	0.252
Q13	0.790	0.154
Q14	0.727	0.356

Thus, the users’ psychological contract with the platforms had a two-dimensional structure. According to the meaning expressed by the items in each factor and referring to the existing psychological contract literature, we named the first factor “transactional psychological contract,” which included users’ perception of platform obligations in each transaction. The second factor was named “relational psychological contract,” which included users’ perception of responsibility for the operation and development of the platform. The platform psychological contract had two dimensions in structure, which was the common feature of all users. However, users can possess different intensities with respect to each item of the psychological contract. In other words, the structure of the psychological contract was homogeneous, but the degree of each item varied by person.

The transactional psychological contract refers to the short-term, specific, economic reciprocal relationship pursued by online ride-hailing platform users. Generally, this contract included two parts. Firstly, users expected to be satisfied by the technical services of the platform, such as the availability and reliability of the app, efficiency of order matching, and accuracy of navigation—which were also the core functions of the platform app. Secondly, users also expected that the platform fees would be fair and reasonable, such as different grades of online ride-hailing pricing. They expected no arbitrary increases in price. Therefore, in each online ride-hailing service, users obtained travel services through the platform app, and the platform received economic benefits through user orders, thus forming a transactional psychological contract. This dimension contained six items.

The relational psychological contract is the users’ higher level of recognition of the platform based on trust, which involves responsibility and commitment beyond the transactional psychological contract. When users believe that they will continue to obtain online ride-hailing services in the future through the platform, they may expect the platform to assume more responsibilities, such as caring about passenger safety, and, thus, hope to maintain a long-term, social, and emotional exchange relationship with the platform. The online ride-hailing platform is an internet-based service enterprise, which is different from the traditional entity-based enterprise—there is no face-to-face service contact between users and the enterprise. Rather, the service contact is only through an app; as a result, the content of the relational psychological contract is different from the previous results based on traditional enterprise research (see Luo, 2006; Wang et al., 2017). Online ride-hailing platforms control a large number of social resources and have become indispensable transportation service providers in China. Therefore, it is reasonable that users expect these enterprises to assume corresponding social responsibilities and obligations and call on them to build a reliable and relational corporate image. This dimension contained seven items in the final scale.

Different from the traditional service enterprises with stores, the biggest feature of platform enterprises is that they depend on an app as the main carrier and tool to provide services and conduct transactions. Regarding transactions, users interact with the platform completely through the app, so some items in the transactional content are specific to the app. Regarding relationships, users interact with the platform continuously through the app. The platform provides all services through an app, and then users and the enterprise establish a long-term relationship. Thus, an app is critical to an internet platform enterprise. For users, the app and the enterprise are one entity.

### Verification Analysis of the Psychological Contract

Confirmatory factor analysis (CFA) was used to further test whether the dimension structure obtained through the EFA could be supported by other sample data. After completing the psychological contract survey, the subjects then answered a list of questions measuring the platform relationship quality.

In this study, 210 valid questionnaires were collected through WENJUANXING^4^, a professional survey website in China. The distribution characteristics of the sample were as follows: in terms of gender, there were 129 female users, accounting for 61.43% of the total sample, and 81 male users, accounting for 38.57%. Respondents between 18 and 40 years old accounted for 92.38% of the total sample. The number of undergraduate/junior college graduates accounted for 85.24% of the sample. Of the total sample, 94.29% used DiDi. One hundred twenty-nine respondents (61.43%) frequently used online ride-hailing services, and 80 (38.1%) used them occasionally. To measure the extent of psychological contract fulfillment, we asked “To what extent do you agree that the platform has fulfilled the following commitments or obligations?,” with a response of “1” indicating “strongly disagree” and “7” indicating “strongly agree.”

**FIGURE 1:**
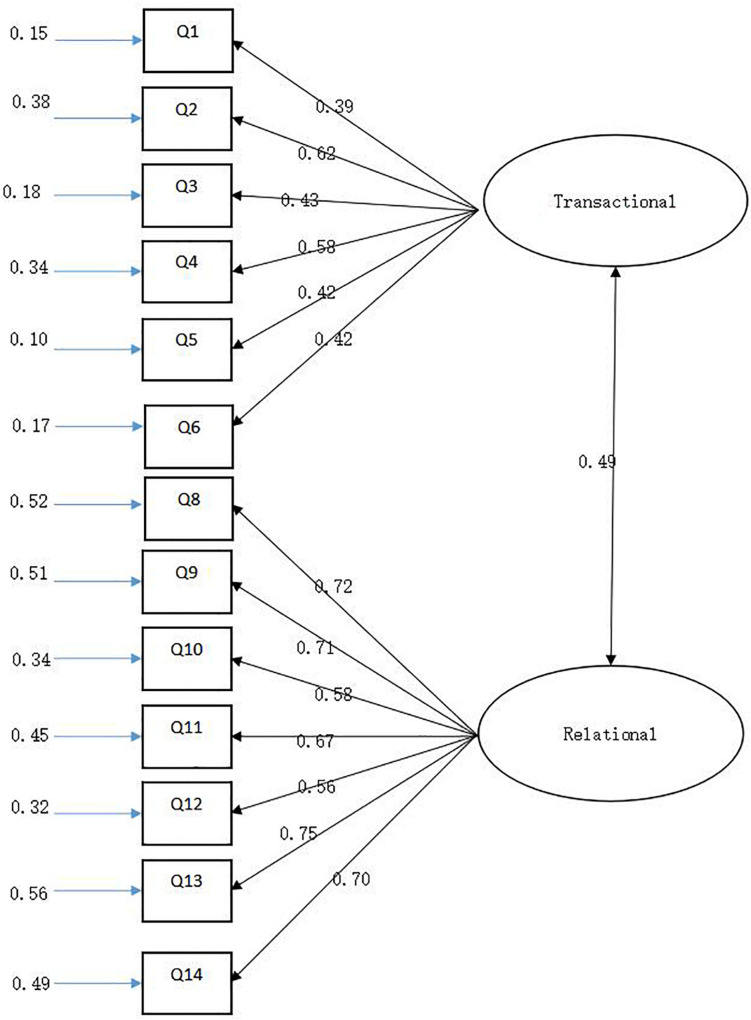
Two-dimensional structure of psychological contract.

Confirmatory factor analysis is a structural model based on existing theories to define a set of related indicators that must be established in advance. We thus used AMOS 20.0 and a structural equation model. The structure of the user psychological contract was verified and analyzed according to the theory established above. The results show that the *T* value of the factor loading of each measurement item was more than 2, with a strong explanatory ability of the factors. Therefore, the users’ psychological contract converges into two factors: transactional and relational psychological contracts. The related model is shown in Figure 1. Finally, the entire model was evaluated, and the indicators of model fitness are shown in Table 3.

**TABLE 3:** Goodness of fit in confirmatory factor analysis.

Indicator	$|\bar{x^{2}/\text{df}}$	*R*	RMESA	GFI	AGFI	IFI	CFI	TLI
Value	2.306	0.115	0.08	0.899	0.857	0.884	0.882	0.856

According to Hou (2002), the numerical range of the fitting index is as follows: $|\bar{x^{2}/\text{df}}$ should be less than or equal to 3; GFI, AGFI, IFI, CFI, and TLI should be greater than 0.9, although a value greater than 0.8 is acceptable; RMESA was between 0.05 and 0.08, indicating that the model was acceptable. Thus, the two-dimensional model of the psychological contract constructed in this paper has an acceptable fitting effect, which shows that the two-dimensional structure of the psychological contract was reasonable.

### Reliability Analysis

To ensure all items have a high degree of consistency in their constructs, the Cronbach’s alpha reliability analysis was conducted. The Cronbach’s alpha coefficient can be used to evaluate the consistency of subjects’ responses to all items. The higher the coefficient of an item, the higher its reliability. Generally, a Cronbach’s alpha greater than 0.6 is acceptable, and one greater than 0.7 indicates high reliability. At the same time, if the deletion of an item increases its internal consistency, then the item should be deleted (Nunnally, 1978). The reliability analysis of the two factors shows that the reliability of the subscale cannot be increased if an item is deleted and the internal consistency of the two factors is greater than 0.60, indicating the acceptable reliability of the scale. The Cronbach’s alphas of the transactional psychological contract and relational psychological contract were 0.636 and 0.850, respectively.

## Effect of Psychological Contract Breach on Platform Relationship Quality

In brand relationship research, scholars have regarded brand relationship quality as an important variable to measure the essence of the relationship between consumers and brands, and define it as the “strength and depth of the relationship between brands and consumers” (Fournier, 1998). Strength refers to the effect of the brand on consumers, while depth emphasizes the frequency of brand–consumer interaction and the level of interdependence. Therefore, the brand relationship quality can be considered an implicit psychological variable reflecting the psychological environment created by the brand relationship (Yao and Liu, 2010). On this account, we will use platform relationship quality as a criterion to test the predictive effect of PCB.

### Psychological Contract Breach and Platform Relationship Quality

A psychological contract is an implicit contract, including one party’s perception of the other party’s obligation to perform (Rousseau, 1990). PCB is the recognition that one party fails to fulfill the obligations and commitments of the other party in the psychological contract. In the interaction, when one party perceives that the other party fails to fulfill its commitments or obligations, it will result in PCB (Morrison and Robinson, 1997). The breach of the psychological contract will bring serious consequences—a psychological contract violation could affect the intention to reuse an online shopping website, for example (Malhotra et al., 2017). In the context of online car hailing, passengers also have psychological contracts for services delivered by the platform. When the passengers perceive that the platform fails to fulfill its obligations, the psychological contract breaks down.

A transactional psychological contract implies that the platform promises specific benefits to the passengers, such as the technical reliability, efficiency, and cost-effectiveness of rides hailed online. This strengthens the belief that passengers can make exchanges with the platform. That is, through the platform, users can obtain fairness in tangible transactions as well as the expected functional value. A breach of a transactional psychological contract will cause users to doubt the ability of the platform, and, thus, impair the quality of the relationship between users and the platform.

A relational psychological contract implies that the platform promises users other utilities in addition to the functional value. These utilities include personal and privacy security, guarantee of service recovery, continuous service improvement, and other social values. The fulfillment of a relational psychological contract will help establish a social link between users and the platform, and directly strengthen and deepen this relationship. In contrast, a breach of the relational psychological contract is negatively related with the quality of the user–platform relationship.

If the developed psychological contract scale is effective, then the PCB measured based on it should predict the relationship quality between users and the platform. Logically speaking, the PCB can affect the quality of the relationship, but the opposite is not true. However, we assume that there are significant negative correlations between PCBs and the platform relationship quality. Therefore, this study used correlation and regression analyses to test the hypothesis.

### Method

#### Measurement of Platform Relationship Quality

The platform relationship quality measurement was based on the brand relationship quality scale proposed by Fournier et al. (2012). We modified the latter scale according to our objective and the characteristics of Chinese consumers. The scale was scored using Likert’s 7-point method, where “1” indicates “strongly disagree” and “7” indicates “strongly agree.”

Brand relationship quality includes six dimensions: partner quality, interdependence, love and passion, personal commitment, intimacy, and self-connection. According to the characteristics of the platform, partner quality and commitment were selected to measure the quality of the brand relationship between users and the platform. The main reason for not using the other four dimensions was to reduce the workload of the questionnaire as much as possible. The constructs of commitment and interdependence are also very suitable for measuring the user–platform relationship quality. For example, commitment refers to the stability of consumers’ attitude toward the relationship. PCB is the recognition that the platform has failed to fulfill the commitments in the psychological contract, which, in turn, would weaken users’ commitment. Besides, Montgomery et al. (2018) found that PCB makes committed consumers turn against their preferred brand by reducing trust. Therefore, there is a strong relationship between PCB and commitment. According to the operational definition of interdependence, this construct measures the user’s functional and psychological dependence on the platform, reflecting the user’s belief that the platform is reliable in fulfilling its commitments and obligations. Therefore, a breach of the psychological contract will certainly damage the user’s dependence on the platform. The specific measurement items are listed in Table 4. In this study, the consistency reliability of the scales was greater than 0.7.

**TABLE 4:** Scale of platform relationship quality.

Dimension	Item
Interdependence *M* = 5.137 SD = 0.935 Alpha = 0.746	1. Using this platform has become a part of my life
	2. I am used to using this online ride-hailing platform
	3. I have become dependent on the online ride-hailing platform for travel
	4. I rely on the convenience offered by the online ride-hailing platform
Commitment *M* = 4.375 SD = 1.099 Alpha = 0.747	1. To use this platform, I would accept a slightly higher price than I pay now
	2. I am very loyal to the online ride-hailing platform
	3. If the online car-booking platform is temporarily unavailable, I will be a little inconvenienced
	4. The online ride-hailing platform is so satisfying that I seldom consider other platforms

#### Sample

In this study, 210 valid questionnaires were collected through WENJUANXING. After completing the psychological contract survey, the latter part investigated the quality of their relationship with the online ride-hailing platform. The distribution of samples has been reported previously.

### Results

#### Correlation Analysis

The relationship between the variables was analyzed by correlation analysis using the Pearson coefficient. The correlation coefficients between PCB and platform relationship quality are shown in Table 5.

**TABLE 5:** Correlation analysis results.

		Interdependence	Commitment
		*M* = 4.559 SD = 1.298	*M* = 3.643 SD = 1.255
Transactional PCB	*M* = 2.794 SD = 0.690	−0.313*	−0.270*
Relational PCB	*M* = 3.846 SD = 1.111	−0.351*	−0.580*

* indicates *p* < 0.01. Psychological contract fulfillment and psychological contract breach have a mutually positive and negative relationship. The positive scoring reflects the degree of psychological contract fulfillment, and the reverse scoring reflects psychological contract breach.

Psychological contract fulfillment and PCB are two sides of the same coin. In the survey, the positive score reflects the fulfillment of the psychological contract, and the negative score reflects the breach of the psychological contract. The results show that both transactional PCB and relational PCB are negatively correlated with interdependence and commitment. In addition, the negative correlation between relational PCB and the platform relationship quality is generally higher in absolute value.

#### Regression Analysis

Next, we took the two dimensions of platform relationship quality as dependent variables for regression analysis. To investigate the effect of different dimensions of PCB, we examined the value-added validity of the two dimensions of PCB in different situations. The results of regression analysis are shown in Table 6.

**TABLE 6:** The regression results of psychological contract breach on platform relationship quality.

Variable	Interdependence		Commitment	
		
	$|\bar{\beta }$	$|\bar{\Delta \,{\text{R}}^{2}}$	$|\bar{\beta }$	$|\bar{\Delta \,{\text{R}}^{2}}$
M1-1: T-PCB	−0.296***	0.127***	−0.264***	0.030*
M1-2: T-PCB++ R-PCB	−0.274***	0.065***	−0.552***	0.266***
M2-1: R-PCB	−0.344***	0.132***	−0.577***	0.028*
M2-2: R-PCB + T-PCB	−0.200**	0.035**	−0.070	0.004

*P < 0.05; **P < 0.01; ***P < 0.001. The values in the table represent $|\bar{\beta }$ and $|\bar{\Delta \,{\text{R}}^{2}}$ corresponding to the last variables added in the regression. For example, the values corresponding to “M1-2: T-PCB+ R-PCB” represent the regression results of relational psychological contract breach with respect to the dependent variables after adding relational psychological contract breach based on M1-1.

Models M1-1 and M1-2 show the regression analysis results of the two dimensions of PCB with respect to the platform relationship quality after controlling for gender and frequency in using online ride-hailing platforms, respectively. The results show that transactional PCB had significant negative effects on interdependence and commitment ($|\bar{\beta }$ = −0.296, *P* < 0.001; $|\bar{\beta }$ = −0.264, *P* < 0.001). Relational PCB also had significant negative effects on interdependence and commitment ($|\bar{\beta }$ = −0.344, *P* < 0.001; $|\bar{\beta }$ = −0.577, *P* < 0.001).

The results of model M1-2 show that when controlling the transactional PCB, the value-added validity of relational PCB with respect to interdependence and commitment was significant $|\bar{\Delta \,{\text{R}}^{2}}$ = 0.065, *p* < 0.001; $|\bar{\Delta \,{\text{R}}^{2}}$ = 0.266, *p* < 0.001). The results of model M2-2 show that when controlling the relational PCB, the value-added validity of transactional PCB with respect to interdependence was significant $|\bar{\Delta \,{\text{R}}^{2}}$ = 0.035, *P* < 0.01), while the value-added validity of commitment was not significant.

## Discussion

This article empirically explored the dimension and content of users’ psychological contract with platform enterprises by using online ride-hailing platforms as an example. The scale developed herein has a clear structure and satisfactory reliability and validity. The data obtained from the questionnaire survey verified the predictive effect of the two dimensions of PCB on platform relationship quality.

### Dimensions of the Psychological Contract With Online Ride-Hailing Platforms

This study designed a questionnaire about the psychological contract of online ride-hailing users. We employed both EFA and CFA to show that the psychological contract between users and the online ride-hailing platforms manifests as transactional and relational psychological contracts. This result is consistent with the existing theoretical and empirical results on psychological contracts (Roehling, 1997). The empirical results also show that there are differences in content and structure between the two dimensions of the psychological contract scale, reflecting the different aspects of the psychological contract. Secondly, we examined the relationship between PCB and the platform relationship quality. The results further showed that the breach of the two dimensions of the psychological contract and platform relationship quality were significantly negative.

The results of this study validate Rousseau’s (1990) psychological contract theory under an employee–employer relationship and Roehling’s (1997) consumer–enterprise relationship. In the current study’s context, our work deepens and expands the psychological contract theory.

### Content of Psychological Contracts With Online Ride-Hailing Platforms

Although we revealed that a psychological contract has a two-dimensional structure, the content of psychological contracts with online ride-hailing platforms is unique. Specifically, users’ relational psychological contract mainly includes the platform’s expected social responsibility, while the content of the relational psychological contract based on traditional enterprises is not seen. We believe this finding is related to the special social responsibility of sharing platforms in China.

#### Social Responsibility of Internet Platform Enterprises

An internet platform is the node of information, capital, and audience flows in virtual space. It is also the projection of a real, social structure in this space. An internet platform must thus meet its economic, legal, and social responsibilities. Therefore, platform economic activity is not only an economic behavior but also a social responsibility behavior.

#### Corporate Social Responsibility of Online Ride-Hailing Platforms

In contemporary society, fulfilling social responsibility has increasingly become a strategic choice for the sustainable development of enterprises. In reality, most enterprises will take, or at least claim to, take some social responsibility actively or passively; online ride-hailing platform enterprises are no exception. However, there is still no consensus on how much social responsibility a platform should undertake at different stages of development. It is not without cost to assume social responsibility, which comes at the expense of either economic interests or the speed of development. As the central entity controlling key information and data, if an online ride-hailing platform evades its due social responsibility, it will seriously damage the interests of consumers and eventually be abandoned. However, as a profit-making enterprise, if the platform takes too much social responsibility, the ultimate cost may still be transferred to all consumers. At present, China’s online ride-hailing platform enterprises are still in a “barbaric growth period.” The platform rules and regulatory systems are not sufficient for this industry. To grow and occupy the market quickly, the platforms have preferred to relax requirements for verification of drivers’ qualifications, resulting in a large number of “problematic drivers.” This practice reflects the platforms’ lack of social responsibility, and the adverse effects of users’ negative experiences and a fear for their personal safety have begun to erode the platform (DiDi is a typical case).

However, it must be noted that the social responsibility of internet platform enterprises is not only an economic problem in the market arena but also a social problem in the public arena. It is impossible to achieve effective governance by relying solely on the platform enterprise. Therefore, it is necessary to strengthen the cooperation among the platform-related subjects (platform, government departments, social organizations, drivers, and passengers) and give full play to the best advantages of each subject in governance to form a cooperative governance network.

#### Public Expectations for Social Responsibility of Platforms

As a light-asset and high-tech enterprise, the online ride-hailing platform forms the entry-point and carrier for numerous users and drivers to trade. China’s largest online ride-hailing platform, DiDi, has a large amount of traffic data, and its policies and mechanisms affect people’s livelihoods. Like WeChat, Amazon, and Alipay, dominant online ride-hailing platforms have introduced immense convenience to people’s lives. Over time, people have begun to critically rely on such services—that is, these platforms have become “too big to fail.” Therefore, in addition to requiring platform enterprises to fulfill transactional psychological contracts, the public also expects the platforms to fulfill social responsibilities, such as improving public transport, ensuring the safety of drivers and passengers, protecting private information, implementing service remedies, and continuously enhancing social well-being. This constitutes the main content of user’s relational psychological contract. If the platform fulfills the relational psychological contract well, user commitment and dependence will increase, which, in turn, would enhance the relationship quality of the platform. With the increasing size and influence of the platforms, the necessity and ability to undertake social responsibility is increasing, as is public expectation that these platforms will fulfill their social responsibility. As a result, the content of social responsibility in psychological contracts will be increasingly stringent.

### Implications

This study has important management implications for sharing platform enterprises within the online ride-hailing service market. From initial praise and user support to recent doubts and dissatisfaction, the dilemma encountered by DiDi fully illustrates the truth that water can both carry a boat and overthrow it. Why have users’ attitudes toward DiDi changed so much? There are various opinions on the internet. Some customers say that such platform enterprises are unable to supervise and ensure the quality of service of its millions of drivers. Others say that DiDi is encumbered by capital and has deviated from its original intention to be a great company. The platform has also been criticized for merely being a medium connecting private car owners and passengers, and that it should not be relational in terms of drivers’ failures. Indeed, according to the platform’s user agreement, the legal liability of the platform is limited. However, in reality, many users view the platform as having almost unlimited responsibility. We find that this contradiction and antagonism reflects the inconsistency between a tangible economic contract and an intangible psychological contract. This tussle is a challenging feature of operating and managing such a platform.

According to our survey results, the degree of fulfillment of relational psychological contracts (*M* = 4.154) is lower than that of transactional psychological contracts (*M* = 5.206). That is, online ride-hailing platforms pay more attention to technology than to social responsibility in the operations process, at least from the user’s point of view. Platforms should pay more attention to satisfying users’ relational psychological contract to avoid the serious consequences of a breach thereof. In the early stages of development, online ride-hailing platforms mainly attract users through subsidies. In reality, this type of user loyalty is not stable and users, over time, swiftly move to competitors. Subsidies cannot be perpetually implemented. If platforms want to consolidate their user base and increase consumer satisfaction at the basic transactional level, they must establish long-term relationships with users and strive to create a relational platform image. Specifically, the platforms must take measures to protect user privacy and safety.

Platforms should strengthen the access verification and supervision of online drivers. The platform risks attracting a poorer quality of drivers if it intentionally or unintentionally lowers the threshold for online cars or relaxes supervision to seize the market share. Such decisions are bound to increase negative events, reduce the overall quality of the drivers, and damage user experience.

Further, platforms should focus on service remediation. Although there is no employment relationship between the drivers and the platform, some passengers tend to think that the platform should bear responsibility for service failures attributed to drivers. There are many types of online car services in China, such as taxis operated by third-party transport companies, special cars operated by enterprise platforms, self-employed common cars, and, the most numerous, ride-sharing cars. The legal responsibilities of the platform differs by service type, and users tend to ignore these differences. As a psychological contract involves subjective beliefs, it is different from an objective legal contract—that is, the former is inevitably one-sided and arbitrary. However, the platform *should* self-impose higher requirements through reforms and continuous improvement to meet stringent user demands. Online ride-hailing platforms can also conduct public relations activities in order to enhance the common understanding of such services if these platforms seek to *revise* some contents of the psychological contract. For example, DiDi had launched a series of public discussions with other platforms, media, and government regulators after its 2018 crisis. Public discussions on service failures are also a joint responsibility of drivers, passengers, and the platform.

Owing to its newness, it would help to clarify the responsibilities of online ride-hailing platforms and form a consensus by involving all stakeholders in discussions and, thus, update users’ psychological contracts.

### Limitations and Future Directions

A psychological contract is bidirectional, and we do not explore passengers’ perceptions of their obligations, which may play an important role in consumer behavior. This is a notable limitation of our study. Further, the platform and driver are two different subjects. However, we do not consider the passengers’ psychological contract with drivers. It is also valuable to follow the evolution of the structure, content, and intensity of passengers’ psychological contracts as the economy and society develop.

Indeed, our subject of analysis—online ride-hailing platforms—is only one type of an internet sharing platform. Hostel sharing platforms (e.g., Airbnb and Tujia) and take-out food platforms (e.g., MEITUAN) are also similar to the former in terms of their business model, operating mechanism, and social responsibility. Our results could be cautiously generalized to these markets with due consideration to their contextual peculiarities.

Finally, the business model of online ride-hailing platforms is similar all over the world, but the perception of users from different cultural backgrounds may differ. Therefore, a cross-cultural study of psychological contracts also merits future discussion.

## Data Availability Statement

The raw data supporting the conclusion of this article will be made available by the authors, without undue reservation.

## Ethics Statement

Ethical review and approval was not required for the study on human participants in accordance with the Local Legislation and Institutional Requirements. Written informed consent from the participants’ legal guardian/next of kin was not required to participate in this study in accordance with the National Legislation and the Institutional Requirements.

## Author Contributions

SS did the theory construction and finished the summary of the article. HX did the data analysis. ZW collected the data. YT did the translation. ZT did the article review. All authors contributed to the article and approved the submitted version.

## Conflict of Interest

The authors declare that the research was conducted in the absence of any commercial or financial relationships that could be construed as a potential conflict of interest.

## Footnotes

1 See http://www.chyxx.com/industry/201806/650300.html.

2 See https://www.iimedia.cn/c810/66802.html.

3 See https://xw.qq.com/amphtml/20180907G2181S00.

4 www.wjx.cn
